# Clinicopathological and prognostic significance of speckle-type POZ protein in cancers: a systematic review and meta-analysis

**DOI:** 10.1186/s12935-021-02329-5

**Published:** 2021-11-27

**Authors:** Yan He, Jun Chen, Xingchen Peng, Yanli Xia, Yonglin Su

**Affiliations:** 1 Department of Biotherapy, Cancer Center, West China Hospital, Sichuan University, Chengdu, 610041 Sichuan People’s Republic of China; 2 Department of Ophalmology, West China Hospital, Sichuan University, Chengdu, 610041 Sichuan People’s Republic of China; 3 College of Food and Biological Engineering, Chengdu University, Chengdu, 610041 Sichuan People’s Republic of China

**Keywords:** Speckle-type POZ protein, Cancer, Biomarker, Meta-analysis

## Abstract

**Background:**

Controversial findings have been reported in the impact of speckle-type POZ protein (SPOP) on clinicopathological features and prognosis in diverse cancers. We conducted this meta-analysis to confirm whether SPOP was an effective biomarker to predict clinical stage, cancer differentiation and survival.

**Methods:**

We searched studies published before June 2021 through Medline, Embase, the Cochrane library register of controlled trials and Wanfang databases. The corrections of SPOP expression with expression disparity, tumor differentiation, clinical stage and survival were analyzed.

**Results:**

Our meta-analysis found that higher expression of SPOP was significantly associated with earlier clinical stage, well differentiation and better overall survival. Subgroup analysis showed that the SPOP expression of adjacent tissue was significantly higher than that in cancer tissues of prostate and liver. However, renal cancer presented improved expression of SPOP in cancer tissue.

**Conclusions:**

SPOP has the potential function to act as a novel and effective biomarker for cancer diagnosis and prognostic stratification.

## Introduction

Speckle-type POZ domain protein (SPOP), a Cullin3-RING ubiquitin ligase adaptor, is found to be expressed in various human tissues. The relative molecular mass of SPOP protein is about 47,000 including 374-amino acid [1]. SPOP contains a typical POZ/BTB domain in N-terminal and a MATH/TRAF domain in C-terminal. It acts as a substrate adapter that the BTB domain can bind to the ubiquitin ligase cullin3 and MATH domain bind to a specific substrate [2, 3]. In general, SPOP as a cullin3 ubiquitin ligase adaptor can specifically recognize and recruit substrate proteins for ubiquitylation and degradation [4].

SPOP is an important molecule that is paid a large amount of attention by researchers in recent years, which plays critical roles during normal development and cancer progress. Zhang et al*.* uncovered that SPOP could promote ubiquitination-mediated programmed death ligand 1 (PD-L1) degradation, leading to decreased PD-L1 levels and increased numbers of tumour-infiltrating lymphocytes to regulate cancer immune surveillance [5]. Dai et al*.* elucidated the tumor suppressor role of SPOP in prostate cancer, in which it promoted the degradation of the bromodomain and extraterminal (BET) proteins to further impact the treatment effectiveness of BET inhibitors [6]. In addition, SPOP might regulate androgen (AR) signaling way and SPOP down-expression led to the activation of AR signaling exerting oncogenic effect in prostate cancer [7]. Thus, SPOP not only can predict cancer prognosis, but also is a novel therapeutic target to affect anti-cancer therapy effectiveness.

As these important roles of SPOP in cancers, increasing evidence pay attention to the relationship between SPOP and tumors in recent years [8]. However, the impact of SPOP on clinicopathological features and prognosis was controversial in these findings. For example, some studies supported that reduced SPOP expression was commonly correlated with a lager tumor size, the present of lymph metastasis and poor differentiation [9]. Nevertheless, several reports had the opposite results. They indicated that SPOP played a tumor-promoting role, and higher SPOP expression was associated with worse clinical stage [10]. Meanwhile, the other studies showed that SPOP expression and tumor size or metastasis of tumor patients were not statistically significant [11, 12]. Due to the small sample size and discrete outcomes, these factors prevented consensus on the role of SPOP. Thus, a meta-analysis to investigate the impact of SPOP on cancers was warranted. Therefore, we performed a meta-analysis to systematically evaluate the value of SPOP in the clinicopathological characteristics and prognosis of patients with cancer using the newest publications.

## Methods

### Data sources and literature search

The reporting of this meta-analysis was based on Preferred Reporting Items for Systematic Reviews and Meta-Analyses (PRISMA). We searched studies published before June 2021 through Medline, Embase, the Cochrane library register of controlled trials and Wanfang databases. The following combined keywords were used in the search: (“speckle-type POZ protein” or “SPOP”) and (“Neoplasms” or “Neoplasia” or “Neoplasias” or “Neoplasm” or “Tumors” or “Tumor” or “Cancer” or “Cancers” or “Malignancy” or “Malignancies”). The reference lists were manually searched to find eligibility records.

### Eligibility criteria

The studies which reported the correlation between SPOP expression and clinical stage or cancer differentiation or overall survival (OS), or compared SPOP expression in cancer and adjacent tissue were included. Studies that were case reports, letters, reviews, animal trials, meeting abstracts were filtered out.

### Data extraction and quality assessment

The following information was extracted from each studies: the name of the first author, publication year, country, sample size, sample source, detection method, cutoff standard, antibody and interesting outcomes. The quality assessment was conducted according to the Newcastle–Ottawa quality assessment scale (NOS). The score ranged from 0 to 9. The NOS score  ≥ 5 suggested that the study was high or moderate quality and it could be included in this meta-analysis.

### Statistical analysis

Risk ratios (RR) were used to assess dichotomous data and hazard ratios (HR) were chosen for survival data with corresponding 95% confidence intervals (95% CI). RR  > 1 indicated that SPOP expression level of the former is higher than the later. Both the random-effect and fixed-effect models were conducted. The value of the inconsistency index (I^2^) was applied to evaluate the heterogeneity among the included studies. If significant heterogeneity was observed (I^2^  ≥  50%), the random-effect modes was used; If not, the results of fixed-effect model was used. Subgroup analyses were based on the cancer type. The robustness of pooled results was assessed by the sensitivity analysis [13]. The potential publication bias was detected by the funnel plot and Egger’s test. P value < 0.05 was considered statistically significant. All statistical analyses were performed by the R software (version 4.0.3, Vienna, Austria). The literature search, record selection, data extraction, quality assessment and statistical analysis were conducted independently by two authors and disagreement was resolved by all authors.

## Results

### Literature search and the study characteristics

**Fig. 1:**
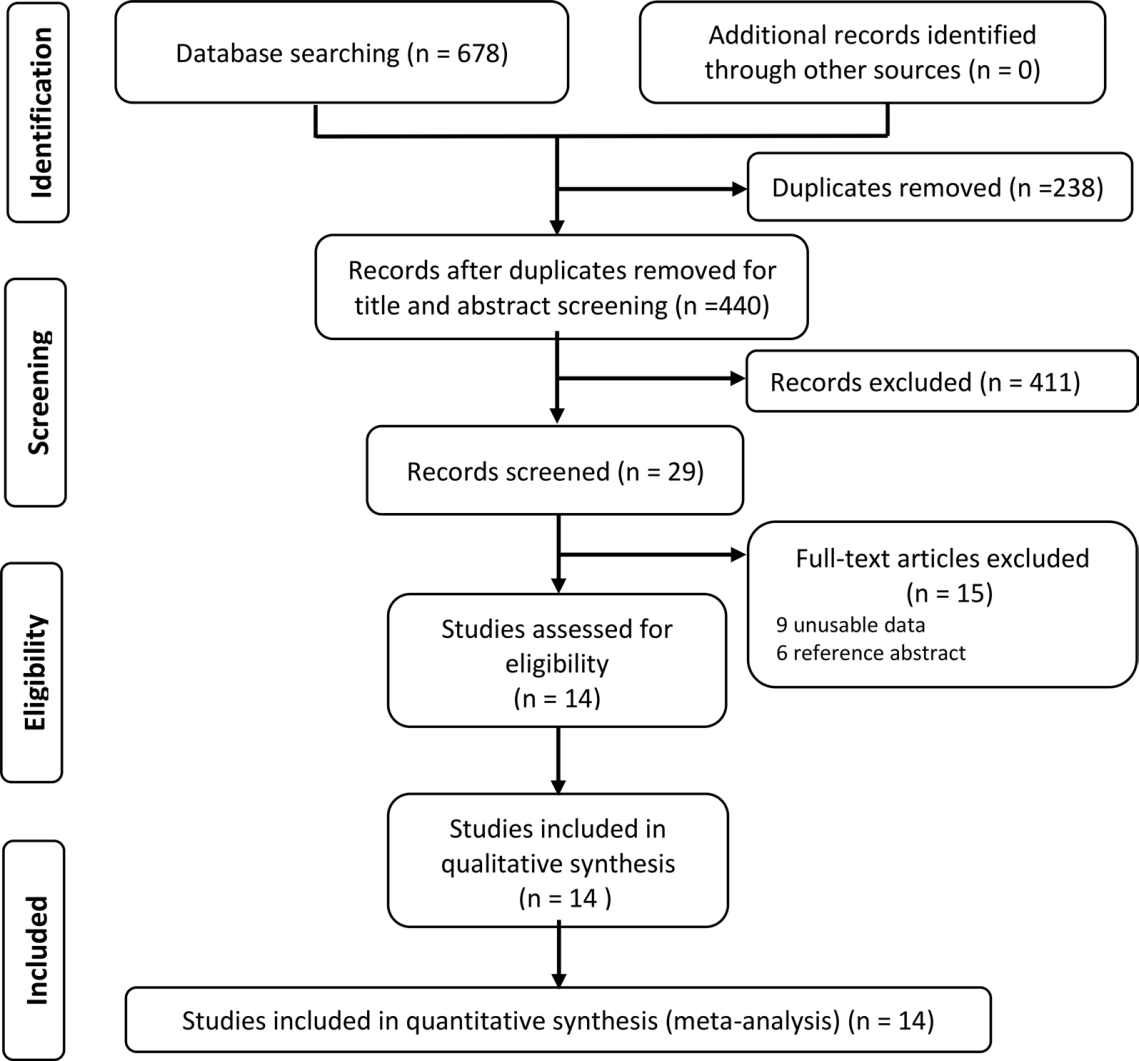
PRISMA flow diagram

A total of 678 records were found after the initial search of Medline, Embase, Cochrane library register of controlled trials and Wanfang databases. Due to duplication, 238 records were removed. Then, titles and abstracts were scanned, and 411 records were excluded according to exclusion criteria. Next, after the evaluation of the full-text articles, 9 records were excluded for the shortage of clinicopathological or prognostic data and 6 records were reference abstracts [14–19]. Finally, as shown in Fig. 1, a total of 14 studies [9–11, 20–30] were eligible for this meta-analysis, which contained 2582 cases from 3 different countries (China, USA, and Korea) and were published from 2009 to 2020. Among them, more than 8 cancer types were investigated, including prostate cancer, ovarian cancer, renal cancer, gastric cancer, liver cancer, lung cancer, glioma and colorectal cancer. Basic characteristics, including first author, publication year, country, sample size, sample source, detection method, cutoff standard, antibody, and NOS score were listed in Table 1.

**Table 1:** Study characteristics

Study ID	Country	Cancer type	Sample size	Sample source	Detection method	Criterion of low expression	Antibody	NOS score
Liu et al. 2017	China	Prostate cancer	70	Tissue	IHC	Negative	abcam	8
Li et al. 2020	China	Ovarian cancer	100	Tissue	IHC	Negative	NA	7
Lu et al. 2012	China	Renal cancer	35	Tissue	IHC	Total score ≤ 2	Abcam (ab81163)	8
Liu et al. 2009	USA	Multiple cancers	1020	Tissue	TMA	Negative	NA	7
Kim et al. 2012	Korea	Multiple cancers	360	Tissue	IHC	˂ 19% of the cells showed	GeneTex (GTX106942)	8
Zeng et al. 2014	China	Gastric cancer	202	Tissue	IHC	Total score ≤ 6	Protein-Tech (16750-1AP)	8
Huang et al. 2015	China	Liver cancer	144	Tissue	IHC	Total score ≤ 3	Santa Cruz (sc‑66649)	8
Li et al. 2019	China	Ovarian cancer	85	Tissue	IHC	Total score ≤ 6	Protein-Tech (16750-1AP)	8
Li et al. 2017	China	Lung cancer	180	Tissue	IHC	Total score ≤ 2	Santa Cruz (sc‑66649)	8
Ding et al. 2015	China	Gliomas	98	Tissue	IHC	Total score ≤ 2	Santa Cruz (sc‑66649)	8
Xu et al. 2017	China	Gastric cancer	60	Tissue	IHC	NA	NA	7
Xu et al. 2015	China	Colorectal cancer	126	Tissue	IHC	Total score ≤ 4	Santa Cruz (sc‑66649)	8
Ji et al. 2018	China	Liver cancer	44	Tissue	IHC	NA	Millipore (MABC565)	7
Zhao et al. 2016	China	Renal cancer	58	Tissue	IHC	Total score ≤ 2	Santa Cruz	8

*IHC* immunohistochemistry; *TMA* tissue micro-array screening; *NOS* the Newcastle–Ottawa quality assessment scale; *NA* not available

### Meta-analysis

**Fig. 2:**
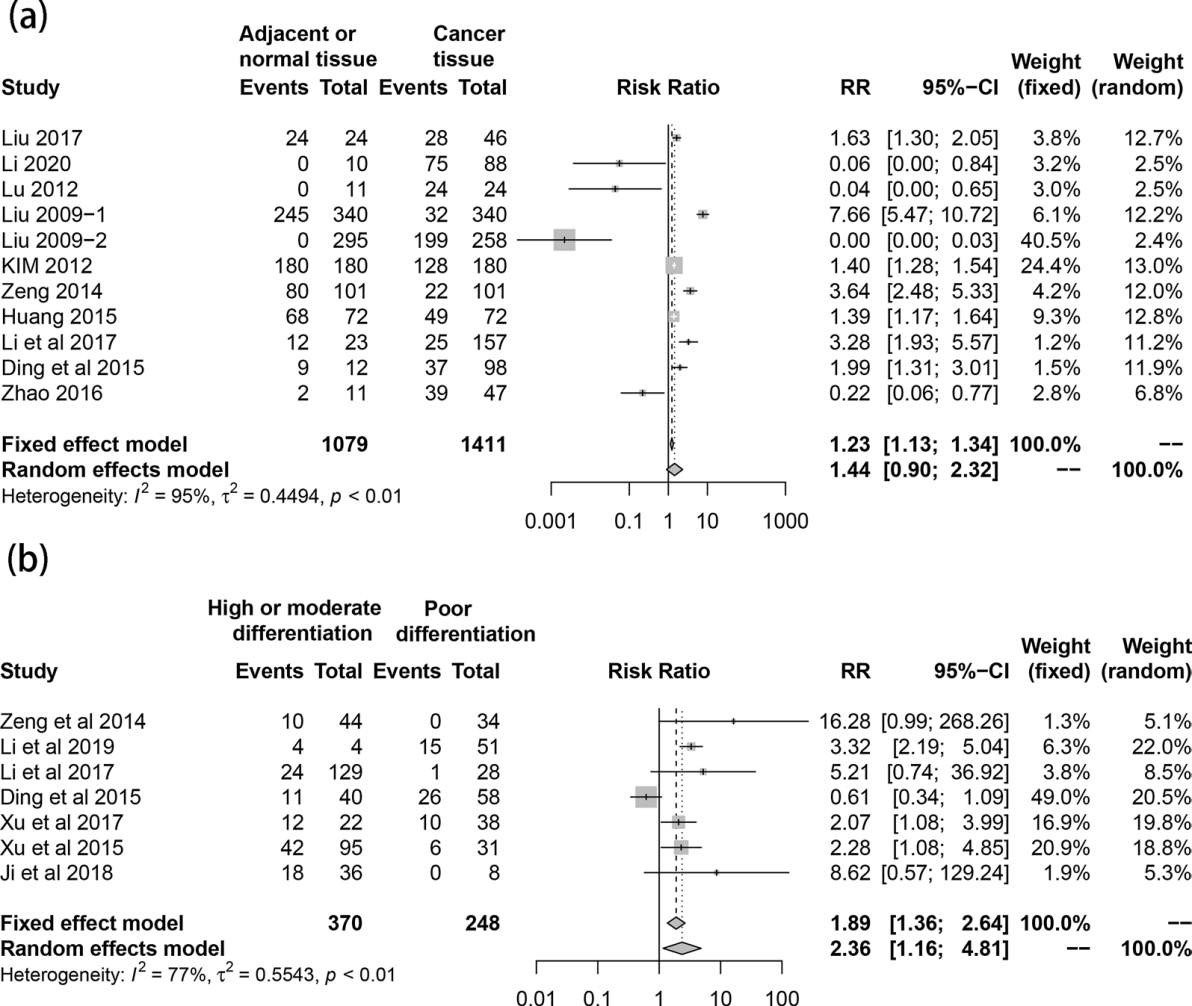
Forest plot. **a** The comparison of SPOP expression in cancer and adjacent or normal tissue. **b** The correlation between SPOP expression and cancer differentiation

**Fig. 3:**
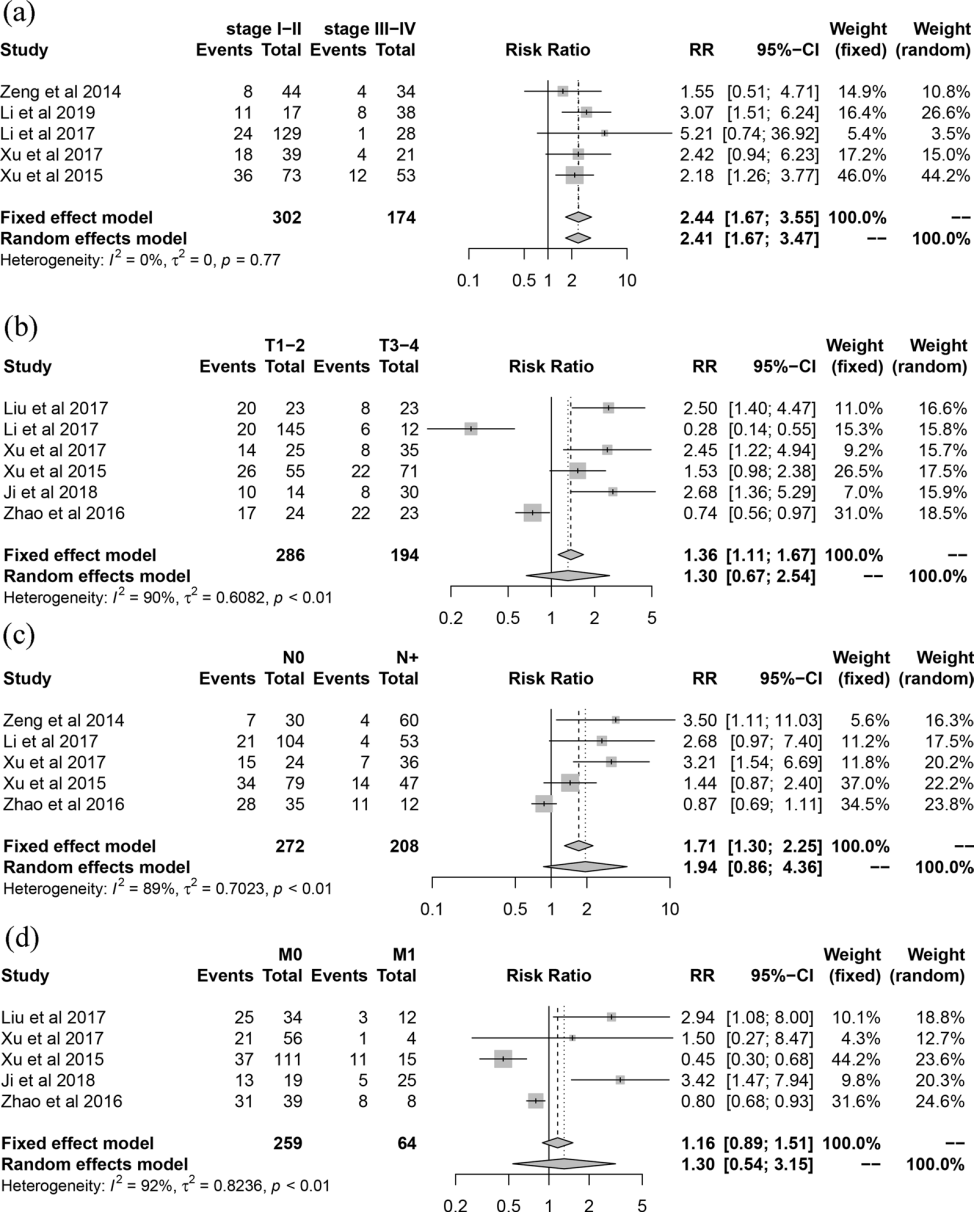
Forest plot of the correlations between SPOP expression and clinical stage (**a**), T classification (**b**), N classification (**c**) and M classification (**d**)

**Fig. 4:**
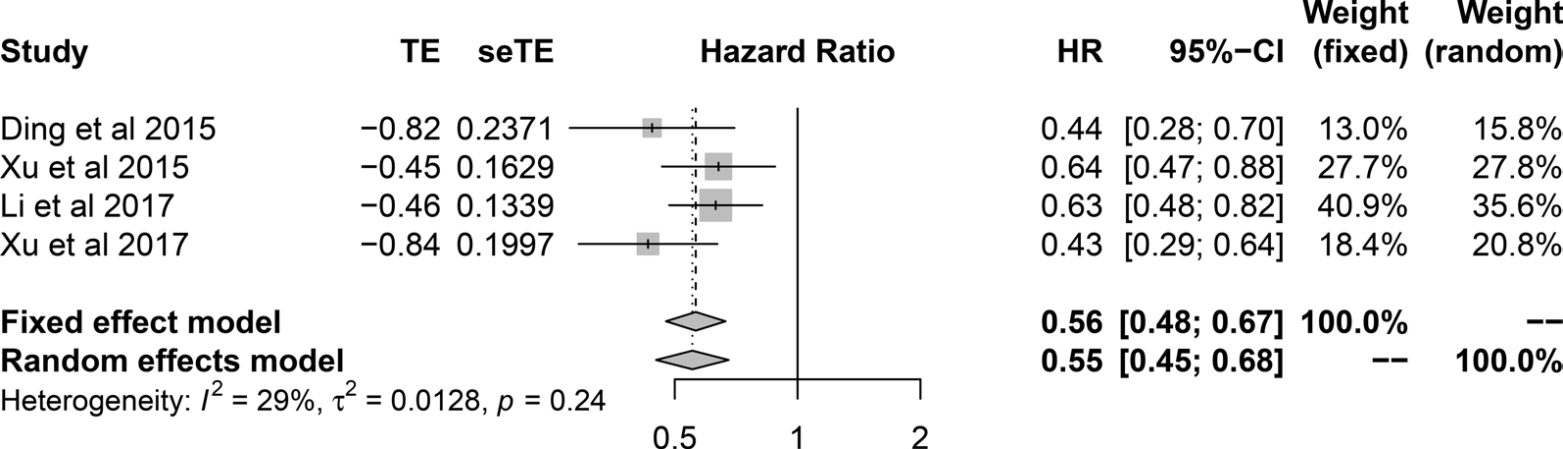
Forest plot of the correlation between SPOP expression and overall survival

In the term of the correlation of SPOP expression with clinicopathological characteristics, SPOP expression was insignificant between cancer and adjacent tissue in total (RR 1.44, 95% CI 0.90–2.32, I^2^  =  95%, random effect model, 11 comparisons, 2490 cases). Pathologically well differentiated tumors were associated with higher SPOP expression level (RR 2.36, 95% CI 1.16–4.81, I^2^  = 77%, random effect model, 7 comparisons, 618 cases) (Fig. 2). Meanwhile, early clinical stage also was statistically associated with higher expression of SPOP (RR 2.41, 95% CI 1.67–3.47, I^2^  = 0%, fix effect model, 5 comparisons, 476 cases). Similarly, although the difference had no statistical significance, in consideration of RR value, larger tumor size (RR 1.30, 95% CI 0.67–2.54, I^2^  = 90%, random effect model, 6 comparisons, 480 cases), more positive lymph node metastasis (RR 1.94, 95% CI 0.86–4.36, I^2^  = 89%, random effect model, 5 comparisons, 480 cases) and distant metastasis (RR 1.30, 95% CI 0.54–3.15, I^2^  = 92%, random effect model, 5 comparisons, 323 cases) showed reduced expression level of SPOP (Fig. 3). Regarding for the correlation of SPOP expression with prognosis, the patients with increased expression of SPOP was statistically associated with better overall survival (HR 0.56, 95% CI 0.48–0.67, I^2^  = 29%, fix effect model, 4 comparisons, 464 cases) (Fig. 4).

### Subgroup analysis

**Fig. 5:**
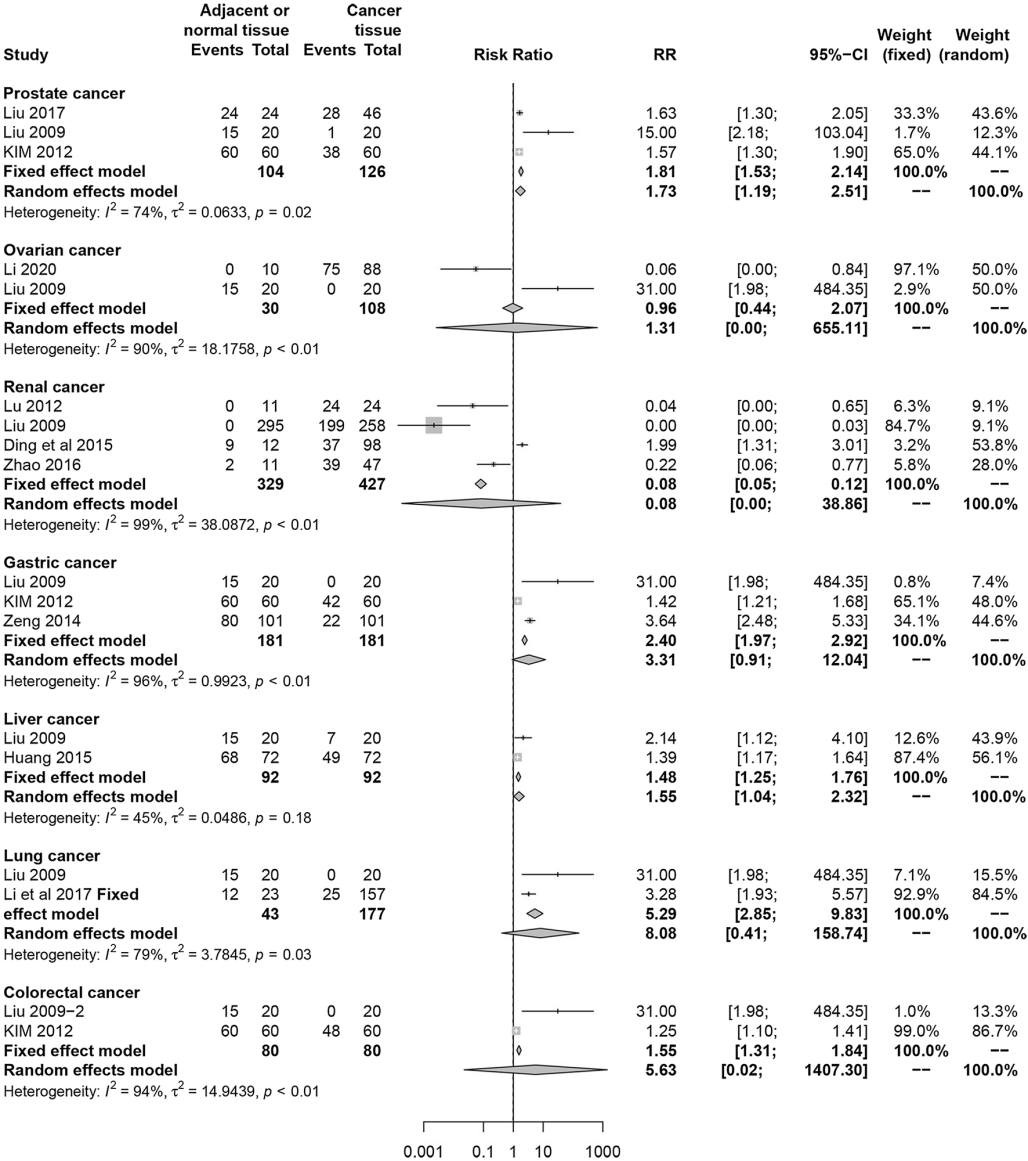
Subgroup analysis of the comparison of SPOP expression in cancer and adjacent or normal tissue

Only cancers that were investigated in above 2 studies were used for the subgroup analysis. As shown in Fig. 5, the SPOP expression of adjacent tissue was significantly higher than that in cancer tissue of prostate (RR 1.73, 95% CI 1.19–2.51, I^2^  = 74%, random effect model, 3 comparisons, 230 cases) and liver cancer (RR 1.48, 95% CI 1.25–1.76, I^2^  = 45%, fix effect model, 2 comparisons, 184 cases). The following cancers showed insignificant difference in SPOP expression level between cancer and adjacent tissue, including ovarian cancer (RR 1.31, 95% CI 0.00–655.11, I^2^  = 90%, random effect model, 2 comparisons, 138 cases), renal cancer (RR 0.08, 95% CI 0.00–38.86, I^2^  = 96%, random effect model, 6 comparisons, 766 cases), gastric cancer (RR 3.31, 95% CI 0.91–12.04, I^2^  = 45%, random effect model, 2 comparisons, 362 cases), lung cancer (RR 8.08, 95% CI 0.41–158.74, I^2^  = 79%, random effect model, 2 comparisons, 220 cases) and colorectal cancer (RR 5.63, 95% CI 0.02–1407.30, I^2^  = 94%, random effect model, 2 comparisons, 160 cases). However, among these cancers, in consideration of RR value or fix effect model, we found that the SPOP expression in adjacent tissue was also higher than that in cancer tissue in gastric cancer (RR 2.40, 95% CI 1.97–2.92, fix effect model), lung cancer (RR 5.29, 95% CI 2.85–9.83, fix effect model) and colorectal cancer (RR 1.55, 95% CI 1.31–1.84, fix effect model). Only renal cancer presented up-regulation of SPOP expression in cancer tissue (RR 0.08, 95% CI 0.05–0.12, fix effect model).

### Sensitivity analysis and publication bias

**Fig. 6:**
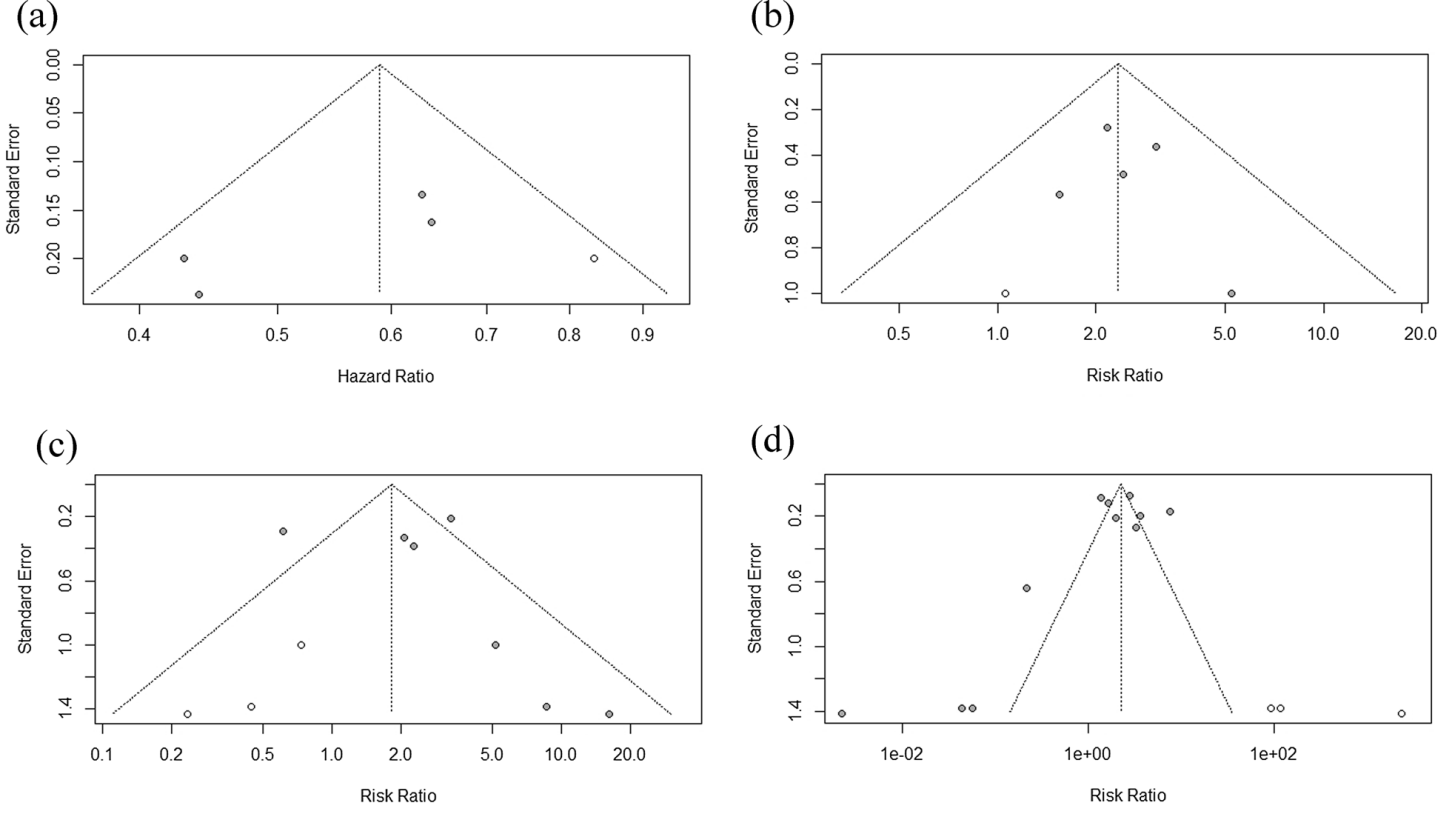
Funnel plot of the correlations between SPOP expression and overall survival (**a**), clinical stage (**b**), cancer differentiation (**c**) and the comparison of SPOP expression in cancer and adjacent or normal tissue (**d**)

Sensitivity analysis was performed for all pooled results by removing the study one by one. No obvious changes were founded in sensitivity analysis when we removed any studies. It indicated that the results of pooled analysis were stable and reliable. Egger’s test was conducted in the SPOP difference expression between cancer tissue and adjacent tissue, and the result indicated no publication bias (P  =  0.98). Similarly, funnel plot showed no publication bias for the meta-analysis of the correction between the expression of SPOP and cancer stage, tissue differential and overall survival (Fig. 6). In conclusion, no obvious publication bias was found in this meta-analysis.

## Discussion

This meta-analysis summarized the clinicopathological and prognosis significance of SPOP expression in cancer patients. Pooled results indicated that up-regulation expression of SPOP was associated with early cancer stage, well differentiation and better overall survival. In addition, SPOP expression of adjacent tissue was significantly higher than that in cancer tissue in prostate and liver cancer. To the best of our knowledge, only one meta-analysis which included 9 studies with 928 patients focused on the association between SPOP expression and prognosis [31]. Because it included studies that must reported OS or progression-free survival, so the comparisons were neglected between SPOP expression and clinicopathological characters. In consideration of the potential limitations to extract survival data from Kaplan–Meier curves, we used data directly obtained from articles for analysis to make our result more dependable. Despite the mentioned above, the results of previous study still supported our findings that compared with patients with lower SPOP expression, patients with higher SPOP expression presented longer overall survival (high versus low expression: HR 0.55, 95% CI 0.38–0.79, P  =  0.001) [31].

Other studies were also given to support our results. Firstly, a previous study demonstrated that the expression of SPOP was higher in adjacent or normal gastric tissues than that in gastric cancer [23]. It was found that the down-regulated SPOP expression was significantly correlated to poor differentiation (P  = 0.013) and advanced clinical stage (P  = 0.002) in colorectal cancer [22]. Another study also revealed that down-regulated SPOP occurred early in prostate tumorigenesis, suggesting that SPOP was an oncogene that could be a predictive marker for prostate cancer [32, 33]. Meanwhile, it was also reported that SPOP played a favorable prognostic factor for liver cancer and might act as a novel tumor suppressor for tumor progression [27]. As mentioned above and based on our research results, down-regulated SPOP expression could predict clinicopathological characters and poor prognosis, suggesting that SPOP protein had the potential to function as prognostic biomarker in cancer patients.

The functions of SPOP in cancer is predominantly dependent on the function of its substrate proteins and the related signaling pathways. It was reported that SPOP expression levels were frequently down-regulated in multiple human tumors and were involved in several signaling pathways [23, 34, 35]. In detail, the loss-of-function mutations of SPOP could prevent ubiquitination-mediated PD-L1 degradation, demonstrating that patients with prostate cancer had a worse prognosis and therapy effect through increasing PD-L1 levels and decreasing tumor-infiltrating lymphocytes [5]. Meanwhile, except for the loss-of-function mutations of SPOP, the hypermethylation of SPOP also led to a decrease in SPOP mRNA and protein levels, suggesting that SPOP was regulated by epigenetic pathways [36, 37]. Notably, SPOP not only can be functionalized as a tumor suppressor by targeting androgen receptor for degradation, but also as an oncoprotein in renal cancer, resulting in activation of androgen receptor driven pathways [38]. Therefore, the potential mechanisms of SPOP in cancers were not fully understood. Understanding the clinical and biological characters of SPOP will lay the foundation and provide a novel view to screen potential targets for precise cancer therapy [38, 39]. Further research on the utility of SPOP as a therapeutic target is also advised. Although early work on the subject reported promising results, there are still few published studies.

Several limitations exist in this meta-analysis. Firstly, the sample sizes of included studies were usually small, which could potentially explain the non-significant findings in subgroup analysis. Secondly, the most of included cases were from Asian, and the results from other populations were required to confirm these corrections. Finally, although we adopted conservative results and explored the sources of heterogeneity, the possible impact on the results due to heterogeneity was not avoid. Therefore, our results need to be further identified in the future.

## Conclusions

This meta-analysis revealed that up-regulation expression of SPOP was associated with early cancer stage, well differentiation and better overall survival. SPOP expression level was insignificant between cancer and adjacent tissue in total. In addition, SPOP expression in adjacent tissue was significantly higher than that in cancer tissue in prostate and liver cancer. The differential expression of SPOP have the potential function to act as a novel and effective biomarker for cancer diagnosis and prognosis.

## Data Availability

Not applicable.

## Abbreviations

SPOP: Speckle-type POZ protein
PD-L1: Programmed death ligand 1
BET: The bromodomain and extraterminal
AR: Androgen
OS: Overall survival
NOS: The Newcastle–Ottawa quality assessment scale
RR: Risk ratios
HR: Hazard ratios
